# Evaluation of an Immunochromatographic Assay as a Canine Rabies Surveillance Tool in Goa, India

**DOI:** 10.3390/v11070649

**Published:** 2019-07-15

**Authors:** Gowri Yale, Andrew D. Gibson, Reeta S. Mani, Harsha P. K., Niceta Cunha Costa, Julie Corfmat, Ilona Otter, Nigel Otter, Ian G. Handel, Barend Mark Bronsvoort, Richard J. Mellanby, Santosh Desai, Vilas Naik, Luke Gamble, Stella Mazeri

**Affiliations:** 1 Mission Rabies, Tonca, Panjim, Goa 403002, India; 2 The Roslin Institute and The Royal (Dick) School of Veterinary Studies, Division of Genetics and Genomics, The University of Edinburgh, Easter Bush Veterinary Centre, Roslin, Midlothian EH25 9RG, UK; 3 Mission Rabies, Cranborne, Dorset BH21 5PZ, UK; 4 National Institute of Mental Health and Neurosciences (NIMHANS), Bangalore 560029, India; 5 Directorate of Animal Health and Veterinary Services, Patto, Panjim, Goa 403001, India; 6 Worldwide Veterinary Service, Hicks ITC, Goa 403507, India; 7 The Royal (Dick) School of Veterinary Studies, Division of Veterinary Clinical Studies, The University of Edinburgh, Hospital for Small Animals, Easter Bush Veterinary Centre, Roslin, Midlothian EH25 9RG, UK

**Keywords:** rabies, rapid, canine, diagnosis, post-mortem, lateral flow, testing, surveillance

## Abstract

Rabies is a fatal zoonotic disease transmitted by the bite of a rabid animal. More than 95% of the human rabies cases in India are attributed to exposure to rabid dogs. This study evaluated the utility of a lateral flow immunochromatographic assay (LFA) (Anigen Rapid Rabies Ag Test Kit, Bionote, Hwaseong-si, Korea) for rapid post mortem diagnosis of rabies in dogs. Brain tissue was collected from 202 animals that were screened through the Government of Goa rabies surveillance system. The brain tissue samples were obtained from 188 dogs, nine cats, three bovines, one jackal and one monkey. In addition, 10 dogs that died due to trauma from road accidents were included as negative controls for the study. The diagnostic performance of LFA was evaluated using results from direct fluorescence antibody test (dFT); the current gold standard post mortem test for rabies infection. Three samples were removed from the analysis as they were autolysed and not fit for testing by dFT. Of the 209 samples tested, 117 tested positive by LFA and 92 tested negative, while 121 tested positive by dFT and 88 tested negative. Estimates of LFA sensitivity and specificity were 0.96 (95% CI 0.91–0.99) and 0.99 (95% CI 0.94–1.00), respectively. The LFA is a simple and low-cost assay that aids in the rapid diagnosis of rabies in the field without the need for expensive laboratory equipment or technical expertise. This study found that Bionote LFA has potential as a screening tool in rabies endemic countries.

## 1. Introduction

Rabies is a fatal zoonotic disease transmitted by the bite of a rabid animal. The rabies virus is endemic across much of the developing world, with free-roaming dogs maintaining epizootic transmission. Successful elimination has been achieved through mass dog vaccination in many settings. However, the disease continues to kill approximately 60,000 people every year, with India carrying approximately one-third of the disease burden at the cost of 2.4 billion USD annually [1]. In recent years, the Indian government has successfully reduced human deaths from other diseases, such as polio, through large scale national public health initiatives. However, efforts to monitor the distribution of the rabies virus and implement effective control measures have remained limited.

The lack of priority for canine rabies control is likely a result of the dearth of data relating to disease burden and distribution. This is due to an absence of routine reporting of suspect rabies cases and insufficient laboratory capacity for rabies diagnosis, as only a few state laboratories have the capacity to test for rabies using either of the World Animal Health Organisation (OIE) approved diagnostic methods, direct fluorescence antibody test (dFT), or real-time PCR (RT-PCR). Effective, wide-reaching surveillance is essential to monitor rabies incidence, vaccination campaign efficacy, and to detect re-incursions to previously rabies-free areas.

Although final laboratory diagnosis using OIE approved techniques is always necessary, there are several potential benefits to the use of an initial screening test that can be performed at the field level. The recent development of immunochromatographic assays, also referred to as lateral flow assays (LFAs), enables immediate testing with limited equipment, infrastructure, and expertise. Recent studies have shown inconsistencies in the specificity and sensitivity of the currently available LFAs, with unacceptable sensitivity variation leading to claims that they are not suitable as a component of rabies surveillance activities [2,3]. This study aims to evaluate the performance of LFA (Bionote) against the dFT gold standard. This is the first large scale study in India to investigate the use of LFA in the diagnosis of rabies in dogs.

## 2. Materials and Methods

### 2.1. Ethics Statement

Brain tissue samples from suspected rabid animals were obtained through routine rabies surveillance program of Mission Rabies and Department of Animal Husbandry and Veterinary Services, Government of Goa. No experiments were done on live dogs. Institutional Animal Care and Use Committee (IACUC) or ethics committee approval was not necessary because moribund animals, dead animals, or animals suspected of rabies were collected by the Goa state rabies surveillance system during routine surveillance and diagnostic service. The authors did not perform any animal sampling during this study. Approval from Veterinary Ethical Review Committee, University of Edinburgh was also obtained; reference number is 80.18 dated 6 August 2018.

### 2.2. Study Area

Goa is the smallest state in India with an area of 3702 km^2^ and a human population of 1,459,000 population density of 490/km^2^ [4]. The state is on the west coast of the country and has a coastline stretching over 100 km [5].

### 2.3. Literature Review

A literature review was conducted to identify previous studies evaluating the diagnostic performance of LFA on experimental or field samples. It was carried out using search engines Google Scholar and PubMed with search terms “rabies, diagnosis, lateral flow assay, immunochromatography”. Searches included any papers published until 18th September 2018. Titles and abstracts were screened to check whether studies included a comparison of the lateral flow assay with the dFT, the gold standard test for rabies diagnosis. Information on study location, numbers of animals tested, species, specificity and sensitivity, were recorded from each publication. Papers that were not published in a peer-reviewed manuscript were also included.

### 2.4. Sample Collection

Mission Rabies and Directorate of Animal Husbandry and Veterinary Services, Government of Goa have been working towards canine rabies elimination from the state through a Memorandum of Understanding (MOU) since 2015 involving mass dog vaccination and rabies surveillance. As part of the MOU with the Government of Goa, Mission Rabies operate a 24/7 telephone hotline publicised for public reporting of aggressive, strangely behaving, rabies suspected dogs. On receipt of a report, a trained team is dispatched to collect the rabies suspect dog. The team was trained in rabies case investigation, including interviews with the reporter, people bitten, witnesses, animal owners and evaluation of the animal. Depending on the findings of the investigation, the dog was either placed in quarantine or humanely euthanised where welfare was compromised. Samples included in this study were collected between October 2016 and April 2018 from various sites in Goa State. All staff members including dog catchers, driver, team leaders, veterinary doctors, veterinary assistants and laboratory personnel were immunised against rabies and antibody levels are checked once in 2 years as a routine health and safety protocol.

Brain tissue was collected by disarticulating the atlanto-occipital joint and using a thick drinking straw through the foramen magnum [6]. Brain tissue was arbitrarily collected without attention to a specific region of the brain. Samples were collected in duplicates; one set for testing immediately by LFA and the other set was stored at −20 °C before being transported to the Rabies diagnostic laboratory, Disease Investigation Unit, Tonca, Goa for dFT and later stored at −80 °C and transported on dry ice in batches to the Department of Neurovirology, National Institute of Mental Health and Neurosciences (NIMHANS), Bangalore, a WHO referral laboratory for rabies diagnosis for dFT proficiency testing.

All samples were tested by LFA at the time of sample collection, and dFT was conducted in batches on all samples. LFA test results were blinded from the dFT investigator.

### 2.5. Lateral Flow Assay (LFA)

**Figure 1:**
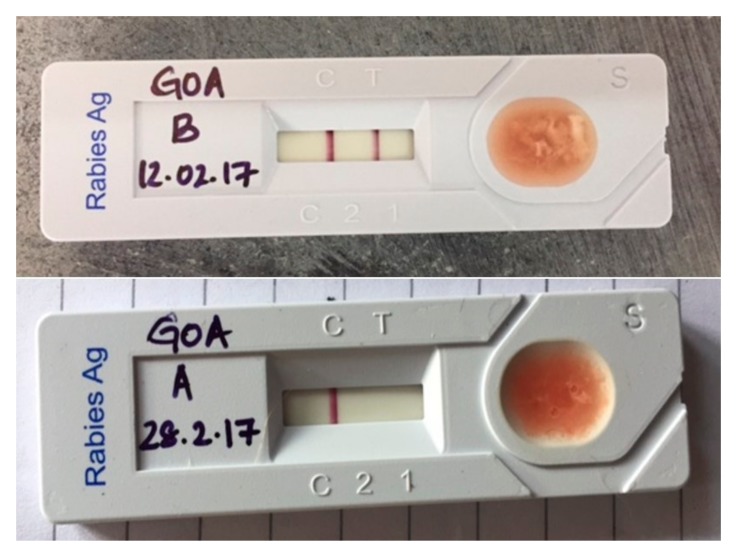
Flow immunochromatographic assay (LFA) showing a positive result (**top**) and a negative result (**bottom**).

The kit used for this study was Anigen, Rapid Rabies Ag Test Kit by produced Bionote, Inc. (Hwaseong-si, Korea). The kit comprises of an LFA device, a plastic pipette and a 1 mL vial with phosphate buffer saline (PBS), encompassing everything required for testing in the field. The procedure was followed according to instructions described in the manual of the kit except for a minor modification of omitting the washing step of the tissue. The brain tissue was directly diluted in media provided in the kit at 1:10 dilution to reduce time and supplies required for testing [2]. The washing step included in the protocol was omitted to simplify the procedure in field conditions as the kit does not provide washing media or PBS. The test takes 10 to 15 min in total, starting from the introduction of brain tissue into PBS vial to bands appearing on the device including waiting for the fluid to flow through the device’s nitrocellulose layer. A single band (at the C position) represents a negative sample, while two lines (one at the C and one at the T position) represent a positive sample. In cases where no line appears the test is considered invalid. The intensity of the bands is not relevant (Figure 1).

### 2.6. Direct Fluorescent (dFT)

Brain tissue samples were subjected to dFT for detection of rabies nucleoprotein antigen using the standard technique accepted by WHO [7]. The technique is based on microscopic examination of impressions or smears of brain tissue after incubation with anti-rabies polyclonal globulin or broadly cross-reactive mAbs (Monoclonal antibodies) conjugated (5100 LIGHT DIAGNOSTICS™ Rabies DFA Reagent) with fluorescein isothiocyanate (FITC). The test is a minimum 2-h procedure which involves brain tissue impression smears on glass slides, fixed in chilled acetone and incubation with FITC conjugate for 45 min before washing to view under a fluorescence microscope (Primostar with LED fluorescent attachment, Carl Zeiss, Weimar, Germany) at 20× and 40× magnification. Time taken to read each slide varies from 5 to 15 min. Positive samples appear with bright apple green fluorescent particles against a dark background. Negative samples appear plain with a dark background.

### 2.7. Statistical Analysis

All data analysis was carried out within the R statistical software environment [8]. LFA, the test under evaluation was compared against dFT, the gold standard test for rabies diagnosis. Diagnostic performance was estimated using package epiR [9]. Package ggplot2 [10] was used for plotting.

## 3. Results

### 3.1. Literature Review

Table 1 presents the details of the performance evaluation of the LFA in field and laboratory conditions in different locations. The sensitivity varied from 32% to 100% and specificity from 98.9% to 100%. The results of the literature review highlight the need for further evaluation of the performance of the LFA in various natural settings encompassing different weather conditions, reservoir species, virus variants and program capacity. Papers that were not peer reviewed were also found which are marked with an asterisk.

### 3.2. Sampled Animal Details

A total of 212 animals were tested; 188 dogs, three bovines, nine cats, one jackal and one monkey were tested for rabies as part of surveillance for the state of Goa. Ten brain samples were collected from dogs that showed no neurological signs but were euthanised due to un-recoverable trauma from automobile road accidents as negative controls for the study. Apart from dogs that were reported to the hotline, Mission Rabies also responded to cases from other NGOs, private veterinary clinics and Government veterinary hospitals. Of the 202 surveillance cases tested, 143 cases were suspected of having rabies, 23 were found dead after showing clinical signs of rabies. 32 animals required euthanasia due to suffering from terminal diseases (Table 2).

### 3.3. Test Results

**Figure 2:**
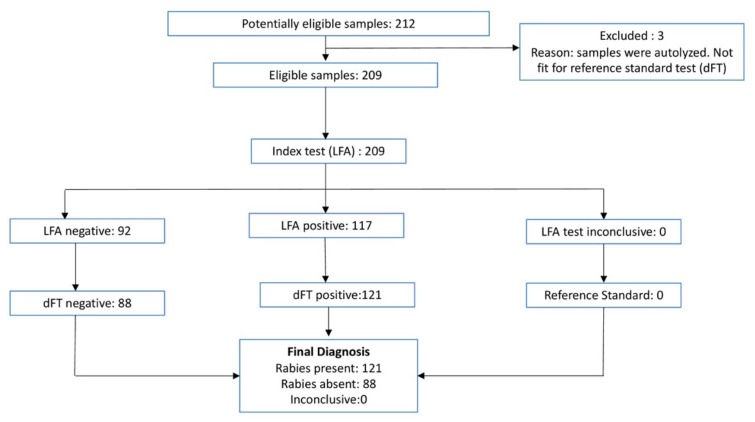
Flowchart showing test results of LFA and direct fluorescence antibody test (dFT).

Test results for each sample tested can be found in Table S1 in the Supplementary Materials. Of the 212 samples tested, three samples could not be read by dFT as they were autolysed due to delay in necropsy and sample collection, so they were removed from the analysis. Of the 209 remaining, 117 samples tested positive and 92 negative by LFA. The same 209 samples tested by dFT resulted in 121 samples positive and 88 samples negative (Table 3, Figure 2). The 10 negative control samples tested negative by both LFA and dFT. Using dFT as a gold standard, the sensitivity of LFA was estimated as 0.96 (95% CI 0.91–0.99) and a specificity of 0.99 (95% CI 0.94–1.00).

The three autolysed samples tested negative by dFT, but results were considered invalid as the samples were autolysed and unfit for diagnosis by dFT. LFA tested negative on two samples but positive on one sample. TaqMan real-time Polymerase Chain Reaction (RT-PCR) was conducted on the three autolysed, results matched the LFA results of two negative and one positive.

## 4. Discussion

This paper presents the results of the first large scale study to investigate the performance of LFA in diagnosing rabies in dogs in India. We compared the results from 209 animals tested both with LFA and dFT. Our results estimated a sensitivity of 96% and specificity of 99%. Three samples received were autolysed and therefore, were not fit for testing by dFT. These samples were tested by both LFA and RT-PCR. Interestingly, one positive LFA result was confirmed by PT-PCR, which might indicate that LFA has the capacity to detect rabies viral antigen in autolysed brain sample where dFT cannot be used.

Historically, dFT on brain tissue was the only ‘gold standard’ test for the confirmation of rabies both in animals and humans, however the World Animal Health Organisation (OIE) recently approved the LN34 PCR and Direct Rapid Immunohistochemistry (DRIT) as other laboratory confirmatory tests for rabies diagnostic test apart from dFT [21,22]. Limitations of dFT include requirements of fresh brain tissue, expensive fluorescent microscope and observer expertise to distinguish specific fluorescence. Apart from reader subjectivity in dFT testing, dFT may not produce reliable results on autolysed or decomposed brain tissue, a frequent challenge encountered in tropical developing countries, such as India, due to high temperatures, lack of refrigeration facilities and considerable transport time involved for a specimen to reach a diagnostic laboratory. Other laboratory diagnostic tests, such as Direct Rapid Immunohistochemistry test (dRIT) and molecular diagnostic tests, perform at equivalent efficiencies as dFT but have similar limitations. The LN34 PCR test has a number of operational advantages over dFT, which offer the potential for more rapid implementation laboratories [23].

A rapid carcass side post mortem test for rabies has the potential to encourage the retrieval and testing of samples from suspect rabies cases in developing countries. Further study is needed to evaluate whether the incorporation of LFA in surveillance activities increases compliance through motivating field operators to take samples. It is possible that the use of LFA would provide additional motivation for testing samples in parts of the country where infrastructure and transport are limited, and therefore, there is no merit in performing the post mortem. Alongside the introduction of LFA, additional focus could be placed on overcoming the challenges of sample transportation and regional laboratory capacity with OIE approved tests. Given the variation reported in the sensitivity of LFA, it is essential that operators are trained to consider negative LFA results as inconclusive until an OIE approved diagnostic test is performed and that human prophylaxis is never influenced by the results of LFA. WHO recently published the third Technical Report Series of Rabies Expert Consultation, where LFA is recommended as a surveillance tool for developing countries lacking adequate laboratory facilities [22].

With the year 2030 being target for elimination of dog-mediated rabies, momentum is building towards canine rabies control in the country [24,25,26], it would be highly recommended to increase rabies laboratory testing facilities in endemic regions and an integrated bite case management approach in low economic countries to help utilise rabies vaccine more efficiently. It would be beneficial to include reliable LFAs as a surveillance tool in the national rabies control plan, to support the expansion of surveillance and testing systems to areas with limited rabies diagnostic capacity. Advantages of the device exceed its limitations in various ways; apart from presenting reasonable sensitivity and specificity, the kit is light, has a shelf life of two years at 25 °C and costs around 5.5 USD per test. The LFA test is not technically difficult to perform and requires limited equipment and infrastructure, enabling field officers to be trained and equipped at low cost. The LFA, apart from being an effective surveillance tool, can also be used for storage and transport of samples for molecular investigations. The device can be archived and also be used for genotyping when required [2].

**Figure 3:**
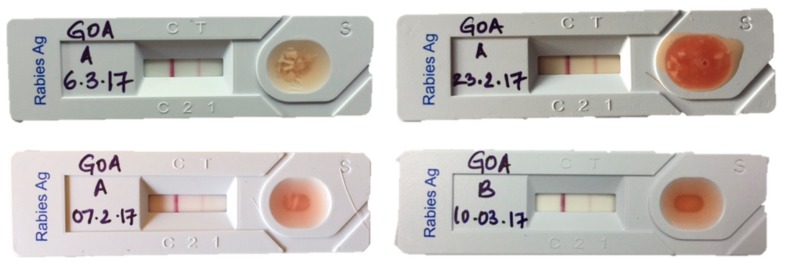
LFAs showing an adequate chromogenic change in the control band and a faint positive line in the test band. These samples were confirmed positive by dFT.

In this study, failure of the LFA to detect five positive samples could be attributed to batch variation in the quality of the product [3] and lack of sufficient virus load. The LFA requires a threshold of antigenic load to bind with antibodies in the test strip for a visible line to appear at the “test” position. Therefore, the sensitivity of the test is likely to decline at low viral loads. Some of the animal cases in this study were in the early stages of rabies and therefore, likely to have lower viral burdens than in the laboratory cases used in other studies [3]. Other limitations include varying intensity of the immunochromatographic test line compared to the control line in positive cases which could be interpreted otherwise if not carefully observed (Figure 3).

## 5. Conclusions

This is the first large scale study to present results on the diagnostic performance of LFA in India. Based on our results, LFA should be considered for use as a practical field tool to support the expansion of surveillance activities in developing countries lacking adequate laboratory facilities. A feasible and efficient surveillance tool would encourage testing and reporting of rabies, leading to better prevention and control efforts.

## Figures and Tables

**Table 1:** Publications on the sensitivity and specificity of Anigen Rapid Rabies Ag Test Kit, Bionote.

Sl.no	Reference	Samples from	Species	Sample Size	No. of Positives	Sensitivity	Specificity	Field/Laboratory
1	Oh JinSik, 2004 * [11]	Korea	Dog, cow, raccoons	75	16	94.1	100	Field
2	Michael, 2005 * [12]	USA	Skunk, bat, horse, dog, cow, cat	200	89	97.7	100	Field
3	Kang, 2007 [13]	Korea	Dog, cattle, raccoon	44	20	91.7	100	Field
4	Australian Animal Health Laboratory 2007 * [14]	Australia	Bats	42	23	100	100	Field
5	Markotter, 2009 [15]	Africa	Canine, bat, jackal, mongoose, feline	25	21	100	100	Laboratory
6	Yang, D.K 2012 * [16]	Korea	Cattle, dog, raccoon	110	20	95	98.9	Field
7	Servat 2012 [17]	Europe	Bats, cats, dogs, foxes, raccoon, mice, lynx, cattle, horse, badger, chinchilla	177	78	88	100	165-Field 12-laboratory
8	Ahmed, 2012 [18]	Sri Lanka, Thailand, Bhutan	Dog, Cow, Cat, human, goat, wild cat, mongoose, grey mongoose, ruddy mongoose, squirrel, rock squirrel, civet cat, rabbit, cow, buffalo, pig, goat, loris, rat, and monkey	503	0	0.74–0.95	0.98–1.0	Field
9	Voehl, 2014 [19]	Africa, Europe, Middle east	Canine, bovine, feline, macaque, porcine, mongoose, equine, jackal, rat, bat	80	32	96.9	100	Field
10	Sharma, 2015 [20]	India	Dog, buffalo, cow, horse, cat	34	24	91.66	100	Field
11	Eggerbauer, 2016 [3]	USA, Azerbaijan, South Africa	Raccoon, bat, dog	102	84	0–100 <= 32	100	51-Field 51-Lab
12	Léchenne, M, 2016 [2]	Chad	Dogs	73	43	95.3	93.3	Field

*: Not peer reviewed.

**Table 2:** Reasons for death/euthanasia of surveillance cases.

Sl.no	Reason for Death/Euthanasia	Total	dFT Negative	dFT Positive	LFA Negative	LFA Positive
1	Suspected for rabies	143	35 *	108	39	104
2	Found dead after showing clinical signs of rabies	23	10 **	13	9	14
3	Canine Distemper	10	10	0	10	0
4	Exposed to a rabid dog	4	4	0	4	0
5	Menace dog	1	1	0	1	0
6	Road Accident	4	4	0	4	0
7	Severe maggot wounds	5	5	0	5	0
8	Sudden death	4	4	0	4	0
9	Suffering	8	8	0	8	0
10	Negative Controls (trauma with no neurological signs)	10	10	0	10	0

*: 1 negative dFT sample was autolysed; **: 2 negative dFT samples were autolysed.

**Table 3:** Cross-tabulation of results of two tests.

	dFT			
**LFA**		**Positive**	**Negative**	**Total**
	**Positive**	**116**	**1**	**117**
	**Negative**	**5**	**87**	**92**
	**Total**	**121**	**88**	**209**

## Supplementary Materials

The following are available online at https://www.mdpi.com/1999-4915/11/7/649/s1, Table S1: Test results for all samples included in study.
