# Supplementary material for: Evaluation of an Immunochromatographic Assay as a Canine Rabies Surveillance Tool in Goa, India
Source: Viruses. 2019 Jul 15;11(7):649. doi: 10.3390/v11070649 (PMC6669590; doi:10.3390/v11070649)
Supplement: Supplementary file 1 [file viruses-11-00649-s001.pdf]

*Table S1: Test results for all samples included in study*

| Sl.no | Case Id | Species | LFT | DIU-FAT | PCR Results |
|---|---|---|---|---|---|
| 1 | Goa-A-22.09.2016 | dog | Negative | Negative | |
| 2 | Goa-A-26.09.2016 | dog | Positive | Positive | |
| 3 | Goa-A-30.09.2016 | dog | Negative | Negative | |
| 4 | GOA-B-8.10.2016 | dog | Negative | Negative | |
| 5 | GOA-A-11.10.2016 | dog | Positive | Positive | |
| 6 | GOA-A-13.10.2016 | dog | Positive | Positive | |
| 7 | GOA-A-14.10.2016 | dog | Negative | Negative | |
| 8 | GOA-A-15.10.2016 | dog | Positive | Positive | |
| 9 | GOA-A-21.10.2016 | dog | Positive | Positive | |
| 10 | GOA-B-21.10.2016 | dog | Negative | Positive | |
| 11 | GOA-A-22.10.2016 | dog | Positive | Positive | |
| 12 | GOA-A-24.10.2016 | dog | Positive | Negative | |
| 13 | GOA-A-26.10.2016 | dog | Negative | Negative | |
| 14 | GOA-B-26.10.2016 | dog | Positive | Positive | |
| 15 | GOA-C-26.10.2016 | dog | Positive | Positive | |
| 16 | GOA-A-28.10.2016 | dog | Negative | Positive | |
| 17 | GOA-A-31.10.2016 | dog | Negative | Negative | |
| 18 | GOA-A-11.11.2016 | dog | Positive | Positive | |
| 19 | GOA-A-14.11.2016 | dog | Positive | Positive | |
| 20 | GOA-A-15.11.2016 | dog | Positive | Positive | |
| 21 | GOA-A-16.11.2016 | dog | Positive | Positive | |
| 22 | GOA-A-24.11.2016 | dog | Negative | Negative | |
| 23 | GOA -A- 30.11.2016 | dog | Positive | Positive | |
| 24 | GOA -A- 11.12.2016 | bovine | Positive | Positive | |
| 25 | GOA -A- 12.12.2016 | dog | Positive | Positive | |
| 26 | GOA -A- 17.12.2016 | dog | Positive | Positive | |
| 27 | GOA -A- 19.12.2016 | dog | Negative | Negative | |
| 28 | GOA -A- 20.12.2016 | dog | Positive | Positive | |
| 29 | GOA -A- 26.12.2016 | dog | Positive | Positive | |
| 30 | GOA -A- 29.12.2016 | dog | Positive | Positive | |
| 31 | GOA -B- 29.12.2016 | dog | Positive | Positive | |
| 32 | GOA -A- 30.12.2016 | dog | Positive | Positive | |
| 33 | GOA -A- 01.01.2017 | dog | Positive | Positive | |
| 34 | GOA -B- 01.01.2017 | dog | Negative | Negative | |
| 35 | GOA -A- 02.01.2017 | dog | Positive | Positive | |
| 36 | Goa-A-05.01.2017 | dog | Negative | Negative | |
| 37 | Goa-B-05.01.2017 | dog | Positive | Positive | |
| 38 | Goa-A-09.01.2017 | dog | Positive | Positive | |
| 39 | Goa-A-10.01.2017 | dog | Positive | Positive | |
| 40 | Goa-B-10.01.2017 | dog | Positive | Positive | |
| 41 | Goa-A-11.01.2017 | dog | Positive | Positive | |
| 42 | Goa-A-12.01.2017 | dog | Negative | Negative | |
| 43 | Goa-D-12.01.2017 | dog | Positive | Positive | |

| | | | | | |
|---|---|---|---|---|---|
| 44 | Goa-A-15.01.2017 | dog | Negative | Negative | |
| 45 | Goa-A-17.01.2017 | dog | Negative | Negative | |
| 46 | Goa-A-19.01.2017 | dog | Negative | Negative | |
| 47 | Goa-A-21.01.2017 | dog | Negative | Positive | |
| 48 | Goa-A-22.01.2017 | dog | Positive | Positive | |
| 49 | Goa-A-23.01.2017 | dog | Negative | Negative | |
| 50 | Goa-A-24.01.2017 | dog | Positive | Positive | |
| 51 | Goa-A-26.01.2017 | dog | Negative | Negative | |
| 52 | Goa-A-28.01.2017 | dog | Negative | Negative | |
| 53 | Goa-A-31.01.2017 | dog | Negative | Negative | |
| 54 | Goa-A-07.02.2017 | dog | Positive | Positive | |
| 55 | Goa-A-11.02.2017 | dog | Positive | Positive | |
| 56 | Goa-A-12.02.2017 | dog | Negative | Negative | Negative |
| 57 | Goa-B-12.02.2017 | dog | Positive | Positive | |
| 58 | Goa-C-12.02.2017 | dog | Positive | Positive | |
| 59 | Goa-A-13.02.2017 | dog | Positive | Positive | |
| 60 | Goa-B-13.02.2017 | dog | Positive | Positive | |
| 61 | Goa-B-14.02.2017 | dog | Positive | Positive | |
| 62 | Goa-B2-14.02.2017 | dog | Negative | Negative | |
| 63 | Goa-A-15.02.2017 | dog | Positive | Positive | |
| 64 | Goa-A-20.02.2017 | dog | Negative | Negative | |
| 65 | Goa-A-23.02.2017 | dog | Positive | Positive | |
| 66 | Goa-A-28.02.2017 | dog | Negative | Negative | |
| 67 | Goa-A-02.03.2017 | dog | Positive | Positive | |
| 68 | Goa-A-04.03.2017 | dog | Positive | Positive | |
| 69 | Goa-B-04.03.2017 | dog | Positive | Positive | |
| 70 | Goa-A-06.03.2017 | cat | Positive | Positive | |
| 71 | Goa-A-10.03.2017 | dog | Positive | Positive | |
| 72 | Goa-B -10.03.2017 | dog | Positive | Positive | |
| 73 | Goa-A-11.03.2017 | dog | Negative | Negative | |
| 74 | Goa-B -11.03.2017 | dog | Negative | Negative | |
| 75 | Goa-C -11.03.2017 | dog | Negative | Negative | |
| 76 | Goa-A-15.03.2017 | dog | Negative | Positive | |
| 77 | Goa-B-16.03.2017 | dog | Positive | Positive | |
| 78 | Goa-C-16.03.2017 | dog | Positive | Positive | |
| 79 | Goa-A-22.03.2017 | dog | Positive | Positive | |
| 80 | Goa-B-22.03.2017 | dog | Positive | Positive | |
| 81 | Goa-A-24.03.2017 | dog | Negative | Positive | |
| 82 | Goa-B-24.03.2017 | dog | Negative | Positive | |
| 83 | Goa-A-29.03.2017 | dog | Positive | Positive | |
| 84 | Goa-A-30.03.2017 | dog | Negative | Negative | |
| 85 | Goa-A-01.04.2017 | dog | Negative | Negative | |
| 86 | Goa-B-01.04.2017 | cat | Negative | Negative | |
| 87 | Goa-A-03.04.2017 | cat | Negative | Positive | |
| 88 | Goa-A-06.04.2017 | dog | Positive | Positive | |
| 89 | Goa-A-09.04.2017 | dog | Negative | Negative | |

| | | | | | |
|---|---|---|---|---|---|
| 90 | Goa-A-18.04.2017 | dog | Positive | Positive | |
| 91 | Goa-B-18.04.2017 | dog | Positive | Positive | |
| 92 | Goa-A-25.04.2017 | dog | Positive | Positive | |
| 93 | Goa-A-27.04.2017 | dog | Negative | Negative | |
| 94 | Goa-A-01.05.2017 | dog | Positive | Positive | |
| 95 | Goa-A-05.05.2017 | cat | Positive | Positive | |
| 96 | Goa-A-16.05.2017 | dog | Positive | Positive | |
| 97 | Goa-A-19.05.2017 | dog | Positive | Positive | |
| 98 | Goa-B-19.05.2017 | dog | Positive | Positive | |
| 99 | Goa-C-19.05.2017 | dog | Positive | Positive | |
| 100 | Goa-E-19.05.2017 | dog | Negative | Negative | |
| 101 | Goa-A-22.05.2017 | dog | Positive | Positive | |
| 102 | Goa-A-23.05.2017 | dog | Positive | Positive | |
| 103 | Goa-A-25.05.2017 | dog | Negative | Negative | |
| 104 | Goa-A-26.05.2017 | dog | Positive | Positive | |
| 105 | Goa-A-03.06.2017 | cat | Negative | Negative | |
| 106 | Goa-A-05.06.2017 | cat | Negative | Negative | |
| 107 | Goa-B-05.06.2017 | dog | Positive | Positive | |
| 108 | Goa-A-06.06.2017 | dog | Positive | Positive | |
| 109 | Goa-B-06.06.2017 | dog | Positive | Positive | |
| 110 | Goa-A-07.06.2017 | dog | Negative | Negative | |
| 111 | Goa-A-12.06.2017 | cat | Negative | Negative | |
| 112 | Goa-A-13.06.2017 | dog | Negative | Negative | |
| 113 | Goa-A-14.06.2017 | dog | Positive | Positive | |
| 114 | Goa-A-15.06.2017 | dog | Positive | Positive | |
| 115 | Goa-A-16.06.2017 | dog | Negative | Negative | Negative |
| 116 | Goa-A-22.06.2017 | dog | Negative | Negative | |
| 117 | Goa-A-24.06.2017 | dog | Positive | Positive | |
| 118 | Goa-A-27.06.2017 | dog | Positive | Positive | |
| 119 | Goa-A-04.07.2017 | dog | Positive | Positive | |
| 120 | Goa-A-11.07.2017 | dog | Negative | Negative | |
| 121 | Goa-A-14.07.2017 | dog | Negative | Negative | |
| 122 | Goa-A-16.07.2017 | dog | Positive | Positive | |
| 123 | Goa-A-21.07.2017 | cat | Negative | Negative | |
| 124 | Goa-A-26.07.2017 | dog | Negative | Positive | |
| 125 | Goa-A-29.07.2017 | dog | Positive | Positive | |
| 126 | Goa-A-30.07.2017 | dog | Positive | Positive | |
| 127 | Goa-A-02.08.2017 | dog | Positive | Positive | |
| 128 | Goa-A-07.08.2017 | dog | Positive | Positive | |
| 129 | Goa-A-10.08.2017 | dog | Positive | Positive | |
| 130 | Goa-A-23.08.2017 | dog | Positive | Positive | |
| 131 | Goa-A-31.08.2017 | dog | Positive | Positive | |
| 132 | Goa-A-05.09.2017 | dog | Positive | Positive | |
| 133 | Goa-B-05.09.2017 | dog | Negative | Negative | |
| 134 | Goa-A-11.09.2017 | dog | Positive | Positive | |
| 135 | Goa-B-11.09.2017 | dog | Positive | Positive | |

| | | | | | |
|---|---|---|---|---|---|
| 136 | Goa-A-13.09.2017 | dog | Negative | Negative | |
| 137 | Goa-A-14.09.2017 | dog | Positive | Positive | |
| 138 | Goa-A- 21.09.2017 | dog | Positive | Positive | |
| 139 | Goa-A-24.09.2017 | bovine | Positive | Positive | |
| 140 | Goa-A-28.09.2017 | dog | Positive | Positive | |
| 141 | Goa-A-07.01.2017 | dog | Negative | Negative | |
| 142 | Goa-B-15.01.2017 | dog | Negative | Negative | |
| 143 | Goa-A-20.04.2017 | dog | Negative | Negative | |
| 144 | Goa-B-10.10.2017 | dog | Negative | Negative | |
| 145 | Goa-A-25.10.2017 | dog | Positive | Positive | |
| 146 | Goa-B-25.10.2017 | dog | Negative | Negative | |
| 147 | Goa-B-29.10.2017 | dog | Negative | Negative | |
| 148 | Goa-A-01.11.2017 | dog | Negative | Negative | |
| 149 | Goa-A-04.11.2017 | dog | Negative | Negative | |
| 150 | Goa-B-04.11.2017 | dog | Negative | Negative | |
| 151 | Goa-A-06.11.2017 | dog | Positive | Positive | |
| 152 | Goa-A-08.11.2017 | dog | Positive | Positive | |
| 153 | Goa-A-15.11.2017 | dog | Positive | Positive | |
| 154 | Goa-B-15.11.2017 | dog | Positive | Positive | |
| 155 | Goa-A-16.11.2017 | dog | Positive | Positive | |
| 156 | Goa-A-21.11.2017 | dog | Negative | Negative | |
| 157 | Goa-A-24.11.2017 | dog | Negative | Negative | |
| 158 | Goa-A-25.11.2017 | dog | Positive | Positive | |
| 159 | Goa-A-29.11.2017 | dog | Negative | Negative | |
| 160 | Goa-A-06.12.2017 | dog | Negative | Negative | |
| 161 | Goa-A-13.12.2017 | dog | Negative | Negative | |
| 162 | Goa-B-15.12.2017 | dog | Positive | Positive | |
| 163 | Goa-A-18.12.2017 | jackal | Positive | Positive | |
| 164 | Goa-A-22.12.2017 | dog | Positive | Positive | |
| 165 | Goa-A-01.01.2018 | dog | Positive | Positive | |
| 166 | Goa-A-02.01.2018 | dog | Positive | Positive | |
| 167 | Goa-B-02.01.2018 | dog | Negative | Negative | |
| 168 | Goa-A-03.01.2018 | dog | Negative | Negative | |
| 169 | Goa-A-04.01.2018 | dog | Positive | Positive | |
| 170 | Goa-A-06.01.2018 | dog | Negative | Negative | |
| 171 | Goa-A-09.01.2018 | dog | Positive | Positive | |
| 172 | Goa-A-10.01.2018 | dog | Positive | Positive | |
| 173 | Goa-A-14.01.2018 | dog | Negative | Negative | |
| 174 | Goa-A-17.01.2018 | bovine | Positive | Positive | |
| 175 | Goa-B-17.01.2018 | dog | Positive | negative | Positive |
| 176 | Goa-A-18.01.2018 | cat | Negative | Negative | |
| 177 | Goa-A-21.01.2018 | dog | Positive | Positive | |
| 178 | Goa-B-21.01.2018 | dog | Negative | Negative | |
| 179 | Goa-A-23.01.2018 | dog | Positive | Positive | |

| | | | | | |
|---|---|---|---|---|---|
| 180 | Goa-A-24.01.2018 | monkey | Negative | Negative | |
| 181 | Goa-A-30.01.2018 | dog | Positive | Positive | |
| 182 | Goa-A-03.02.2018 | dog | Positive | positive | |
| 183 | Goa-A-06.02.2018 | dog | Negative | negative | |
| 184 | Goa-A-16.02.2018 | dog | Positive | positive | |
| 185 | Goa-A-19.02.2018 | dog | Positive | positive | |
| 186 | Goa-A-26.02.2018 | dog | Positive | positive | |
| 187 | Goa-A-27.02.2018 | dog | Negative | Positive | |
| 188 | Goa-A-04.03.2018 | dog | Positive | positive | |
| 189 | Goa-B-04.03.2018 | dog | Positive | positive | |
| 190 | Goa-A-05.03.2018 | dog | Negative | negative | |
| 191 | Goa-B-05.03.2018 | dog | Negative | negative | |
| 192 | Goa-C-05.03.2018 | dog | Negative | negative | |
| 193 | Goa-A-14.03.2018 | dog | Negative | negative | |
| 194 | Goa-A-17.03.2018 | dog | Positive | positive | |
| 195 | Goa-A-26.03.2018 | dog | Negative | negative | |
| 196 | Goa-A-28.03.2018 | dog | Negative | negative | |
| 197 | Goa-A-05.04.2018 | dog | Positive | positive | |
| 198 | Goa-A-18.04.2018 | dog | Negative | negative | |
| 199 | Goa-B-18.04.2018 | dog | Negative | negative | |
| 200 | Goa-A-22.04.2018 | dog | Positive | positive | |
| 201 | Goa-A-26.04.2018 | dog | Positive | Positive | |
| 202 | Goa-A-28.04.2018 | dog | Negative | Negative | |
| NEGATIVE CONTROLS | | | | | |
| 1 | Goa -A- 06/10/2016 (NC 4) | dog | Negative | Negative | |
| 2 | Goa-NC-06.10.2016 (9) | dog | Negative | Negative | |
| 3 | Goa-NC-06.10.2016 | dog | Negative | Negative | |
| 4 | G-NC-06.10.2016 (7) | dog | Negative | Negative | |
| 5 | G-NC-06.10.2016 (3) | dog | Negative | Negative | |
| 6 | G-NC-06.10.2016 (8) | dog | Negative | Negative | |
| 7 | G-NC-06.10.2016 (1) | dog | Negative | Negative | |
| 8 | G-NC-06.10.2016 (2) | dog | Negative | Negative | |
| 9 | G-NC-06.11.2016 (6) | dog | Negative | Negative | |
| 10 | Goa -B- 26/12/2016 (NC) | dog | Negative | Negative | |
